# Lipopolysaccharide structure modulates cationic biocide susceptibility and crystalline biofilm formation in *Proteus mirabilis*

**DOI:** 10.3389/fmicb.2023.1150625

**Published:** 2023-04-05

**Authors:** O. E. Clarke, H. Pelling, V. Bennett, T. Matsumoto, G. E. Gregory, J. Nzakizwanayo, A. J. Slate, A. Preston, M. Laabei, L. J. Bock, M. E. Wand, K. Ikebukuro, S. Gebhard, J. M. Sutton, B. V. Jones

**Affiliations:** ^1^Department of Life Sciences, University of Bath, Bath, United Kingdom; ^2^Department of Biotechnology and Life Sciences, Tokyo University of Agriculture and Technology, Tokyo, Japan; ^3^United Kingdom Health Security Agency, Salisbury, United Kingdom

**Keywords:** *Proteus mirabilis*, biocide, biofilm, lipopolysaccharide, catheter associated urinary tract infection, biocide resistance, antimicrobials, AMR

## Abstract

Chlorhexidine (CHD) is a cationic biocide used ubiquitously in healthcare settings. *Proteus mirabilis*, an important pathogen of the catheterized urinary tract, and isolates of this species are often described as “resistant” to CHD-containing products used for catheter infection control. To identify the mechanisms underlying reduced CHD susceptibility in *P. mirabilis*, we subjected the CHD tolerant clinical isolate RS47 to random transposon mutagenesis and screened for mutants with reduced CHD minimum inhibitory concentrations (MICs). One mutant recovered from these screens (designated RS47-2) exhibited ~ 8-fold reduction in CHD MIC. Complete genome sequencing of RS47-2 showed a single mini-Tn*5* insert in the *waaC* gene involved in lipopolysaccharide (LPS) inner core biosynthesis. Phenotypic screening of RS47-2 revealed a significant increase in cell surface hydrophobicity and serum susceptibility compared to the wildtype, and confirmed defects in LPS production congruent with *waaC* inactivation. Disruption of *waaC* was also associated with increased susceptibility to a range of other cationic biocides but did not affect susceptibility to antibiotics tested. Complementation studies showed that repression of *smvA* efflux activity in RS47-2 further increased susceptibility to CHD and other cationic biocides, reducing CHD MICs to values comparable with the most CHD susceptible isolates characterized. The formation of crystalline biofilms and blockage of urethral catheters was also significantly attenuated in RS47-2. Taken together, these data show that aspects of LPS structure and upregulation of the *smvA* efflux system function in synergy to modulate susceptibility to CHD and other cationic biocides, and that LPS structure is also an important factor in *P. mirabilis* crystalline biofilm formation.

## Introduction

Biocides, a class of antimicrobial chemical agents including disinfectants and antiseptics, are used extensively in healthcare settings. These antimicrobials are integral to strategies aimed at reducing antibiotic use through enhanced infection control (Maillard, 2005). Accordingly, biocide use in clinical settings is increasing, particularly for prophylactic decolonization of newly admitted patients (Gilbert and Moore, 2005; Wand et al., 2017, 2019; Hardy et al., 2018). This raises important questions about the impact of increased biocide use on bacterial pathogens, and there is evidence that sub-lethal biocide exposure can select for problematic species, reduced biocide susceptibility, and other undesirable traits (Hayden et al., 2015; Hardy et al., 2018; Wand et al., 2019). In particular, there is increasing concern that inappropriate, or overuse, of biocides could lead to the emergence of “resistant” strains and select for cross-resistance to antibiotics (Wand et al., 2017; Hashemi et al., 2019; Stein et al., 2019). However, the mechanisms underpinning acquisition of reduced biocide susceptibility in bacterial pathogens remain poorly understood.

Among the most commonly used biocides in healthcare settings is chlorhexidine (CHD; Russell, 1986; Gilbert and Moore, 2005; Kampf, 2016). CHD is a membrane active cationic biocide, which interacts with the negatively charged Gram negative cytoplasmic membrane, bridging adjacent phospholipids and destabilizing the membrane, causing cellular leakage (Gilbert and Moore, 2005). This compound is incorporated in numerous antiseptic and disinfectant products available on the NHS supply chain, with in-use concentrations varying from 0.02 % in bladder washouts and wound irrigation solutions, to 5 % in surgical scrubs (Bock et al., 2016). The wide range of in-use concentrations means that pathogens are likely to be frequently exposed to sub-lethal concentrations that can potentially select for tolerance or other undesirable traits such as increased virulence and cross-resistance to antibiotics (Bock et al., 2016; Wand et al., 2017; Stein et al., 2019; Pelling et al., 2019a). Furthermore, recent studies have demonstrated that common pathogens can adapt to become less susceptible to biocides such as CHD (Wand et al., 2017; Hardy et al., 2018; Pelling et al., 2019a; Sethi et al., 2021).

Of note in this regard is the low CHD susceptibility often observed in isolates of *Proteus mirabilis.* This organism is an important pathogen of the catheterized urinary tract, associated with up to 44 % of catheter-associated urinary tract infections (CAUTIs; Schaffer and Pearson, 2015). The ability of *P. mirabilis* to block urethral catheters, through the formation of extensive crystalline biofilms on catheter surfaces, is associated with serious complications in individuals undergoing long-term urethral catheterization (Loveday et al., 2014; Armbruster et al., 2018). A range of products containing CHD are available for catheter infection control, including bladder irrigation solutions containing 0.02 % *w*/*v* CHD (200 μg mL^−1^; Bock et al., 2016). However, *P. mirabilis* clinical isolates often exhibit greater CHD MICs (256 -≥ 512 μg mL^−1^), and associated recalcitrance to catheter management products containing CHD. As such, this organism is often described as chlorhexidine “resistant” or “tolerant” (Gillespie et al., 1967; Stickler, 1974; Kampf, 2016; Pelling et al., 2019a).

Our previous work highlighted the *smvAR* efflux system as an important factor in *P. mirabilis* CHD tolerance (Pelling et al., 2019a). The clinical isolate RS47 (CHD MIC ≥512 μg mL^−1^) was shown to harbor mutations in the *smvR* repressor which results in overexpression of the cognate SmvA efflux pump. Restoration of SmvR activity in RS47 reduced the CHD MIC ~2-fold, confirming the contribution of *smvA* efflux to the CHD tolerant phenotype of this isolate (Pelling et al., 2019a). However, the CHD MIC of *smvR* complemented RS47 still remained notably higher than for the most CHD susceptible *P. mirabilis* clinical isolates we have characterized to date (CHD MICs of 8–16 μg mL^−1^). This indicated that *smvA* overexpression was not the only factor underpinning the high CHD MIC of RS47. Here we employed random transposon mutagenesis to identify additional mechanisms underpinning CHD “resistance” in *P. mirabilis*, and elucidate the synergistic effects of LPS structure and efflux in modulating CHD susceptibility. In doing so we also highlight the role of LPS structure in crystalline biofilm formation and blockage of urethral catheters by *P. mirabilis.*

## Materials and methods

### General culture and media

Clinical isolates of *P. mirabilis* were obtained from The Royal Sussex County Hospital (Table 1). Isolates were routinely cultured in Lysogeny Broth (LB; 10 g L^−1^ tryptone, 5 g L^−1^ yeast extract, 10 g L^−1^ sodium chloride) or Tryptic Soy Broth (TSB; 17.0 g L^−1^ pancreatic digest of casein, 3.0 g L^−1^ enzymatic digest of soya bean, 5.0 g L^−1^ sodium chloride, 2.5 g L^−1^ dipotassium hydrogen phosphate, 2.5 g L^−1^ glucose) overnight at 37°C with aeration unless stated otherwise. For enumeration of single colonies on solid media, LB agar without salt (NSLB; 2 % *w*/*v* agar) or MacConkey agar without salt (1.5 % *w*/*v* agar) were used. Strains requiring antibiotic selection were cultured in media supplemented with either 50 μg mL^−1^ kanamycin (Km50) or 100 μg mL^−1^ ampicillin (Amp100). All reagents, chemicals and growth media were purchased from Fisher (Thermo Fisher Scientific, United States) or Merck (Merck Group, Germany) unless stated otherwise.

**Table 1:** Strains and derivatives used in this study.

Species/strains	Description	Routine selection	Source
*Proteus mirabilis*			
RS47	Clinical isolate from urinary tract infection. Low CHD susceptibility (CHD MIC > 512 μg mL^−1^). Mutation in *smvR* leading to inactivation and overexpression of *smvA* efflux system.	N/A	Royal Sussex County Hospital (Pelling et al., 2019a)
RS47-2	Mini-Tn*5* mutant of RS47 with Tn insertion in *waaC* gene.	Kanamycin 50 μg mL^−1^	This study
RS47::pGEM-Tempty	Derivative of RS47 harbouring empty pGEM-T vector.	Ampicillin 100 μg mL^−1^	Pelling et al., (2019a)
RS47::pGEM-T*smvR*	Derivative of RS47 harbouring functional s*mvR* cloned in pGEM-T vector.	Ampicillin 100 μg mL^−1^	Pelling et al., (2019a)
RS47-2::pGEM-Tempty	Derivative of RS47-2 mini-Tn*5* mutant harbouring empty pGEM-T vector.	Ampicillin 100 μg mL^−1^	This study
RS47-2::pGEM-T*smvR*	Derivative of RS47-2 mini-Tn*5* mutant harbouring functional s*mvR* cloned in pGEM-T vector.	Ampicillin 100 μg mL^−1^	This study
*Escherichia coli*			
BW29427	Auxotrophic donor strain harbouring pRL27::mini-Tn5 plasmid transferred to RS47 *via* conjugation to generate transposon mutants. Requires supplementation of media with diaminopimelic acid for growth.	Kanamycin 50 μg mL^−1^	Larsen et al. (2002)
JM109	Standard cloning strain, used as an intermediate host for pGEM-T constructs.	Ampicillin 100 μg mL^−1^	Promega, United Kingdom

### Determination of minimum inhibitory concentration

Susceptibility to biocides and antibiotics was evaluated by determination of minimum inhibitory concentrations (MICs) using a 96-well plate (Corning Incorporated) broth microdilution method developed by the UK Health Security Agency (UKHSA) (Bock et al., 2018). Doubling dilutions of antimicrobials were added to wells before inoculation with *ca.* 10^5^ CFU mL^−1^ of overnight culture. Plates were incubated statically for 20 h at 37°C before OD_600 nm_ was measured with a plate reader (Multiskan^™^ FC Microplate Reader). For each compound assayed, the MIC was defined as the lowest concentration which prevented measurable growth.

### Random transposon mutagenesis

Transposon mutagenesis of *P. mirabilis* RS47 was conducted using the pRL27::mini-Tn*5* system as previously described (Larsen et al., 2002; Nzakizwanayo et al., 2015). Vector pRL27 was transferred to RS47 from the diaminopimelic acid (DAP) auxotroph *pir*^+^ donor *E*. *coli* BW29427 by conjugal transfer. Mating experiments were performed using overnight cultures at donor/recipient ratio of 1/10, and mixed cultures were incubated for 8 h at 37°C on NSLB agar supplemented with 100 nM DAP and 10 mM MgSO_4_. Trans-conjugant RS47 colonies were selected and subsequently cultured on NSLB agar supplemented with 50 μg mL^−1^ kanamycin. Individual transconjugants were picked and used to inoculate individual wells of 96-well microtiter plates containing LB broth with 50 μg mL^−1^ kanamycin. Plates were then incubated overnight at 37°C, and cultures were supplemented with sterile glycerol to a final concentration of 10 % and stored at –80°C until required for screenings.

### Mutant screening

Using a 96-pin inoculator, trans-conjugants were gridded onto NSLB agar supplemented with CHD at 0.5 × the wildtype MIC (512 μg mL^−1^), and in parallel gridded onto NSLB agar plates without selection as a positive control. After 48 h incubation at 37°C, the growth of each mutant on plates with and without CHD selection was assessed. Those unable to grow on agar supplemented with CHD, but showing normal growth on NSLB agar alone, were selected for further characterization. To confirm changes in MIC relative to the RS47 WT, overnight cultures of mutants recovered from screens were adjusted to OD_600 nm_ 0.1 and 100 μL aliquots transferred to a 96 well microtiter plates, along with adjusted cultures of the RS47 WT, and gridded onto NSLB agar supplemented with CHD at concentrations ranging from 0 to 512 μg mL^−1^. Growth was recorded after 24 h of incubation at 37°C and mutants exhibiting a stable phenotype and reduced MIC relative to the RS47 WT were retained for further characterization. For mutants selected in this way CHD MICs were then determined using broth microdilution assays.

### Identification of genes disrupted in RS47 mini-Tn*5* mutant of interest

To confirm single copy mini-Tn*5* insertion and identify genes disrupted in one particular mutant showing a significant reduction in CHD susceptibility (designated RS47-2), the complete genome sequence was obtained using the MinION platform and compared to the RS47 WT sequence (Bioproject Accession Number: PRJNA554808). RS47-2 genomic DNA was extracted using the Wizard^®^ Genomic DNA Purification kit (Promega), and DNA libraries were prepared for MinION sequencing using a ligation sequencing kit 1D (SQK-LSK109, Oxford Nanopore Technologies) as per manufacturer’s instructions, with *ca.* 1.5 μg of starting DNA. For sequencing, SpotON flow cells were primed and loaded as per manufacturer’s instructions and run using a MinION MK 1B and MinKNOW software version 1.7 for Mac OS X. Sequence reads were extracted from FAST5 files to FASTA format using poretools software version 0.6.0 (Loman and Quinlan, 2014). Reads ranging from 3 to 70 kb were assembled in Canu version 1.5 (Koren et al., 2017). Default Canu settings were used with a predicted genome size of 4 Mb and adjusted error correction of 0.144. Assemblies were annotated using Glimmer implemented in Geneious version 10.1.3. Regions homologous to the pRL27::mini-Tn*5* were identified using BLASTn in Geneious (at 100 % identity and query coverage, *E*-value of 1 × 10^5^; Altschul et al., 1990; Zhang et al., 2000). Sequences flanking the mini-Tn*5* insert were mapped to the parental RS47 genome to identify the disrupted ORF. To confirm the predicted mini-Tn*5* insert site in RS47-2, primers WAAC-FLANKING-F (5′-CCGCAAATCCAAAGTGGACA-3′) and WAAC-FLANKING-R (5′-TTACTCGCTACGGAGCCATC-3′) were used to amplify the region of interest in the RS47 WT and RS47-2 genomes. Reactions were performed in total volumes of 25 μL using the Qiagen Taq core kit according to manufacturer’s instructions, with 20 ng total DNA template using 15 pmol per total reaction of each primer. The thermal cycler was programmed for one 5 min cycle at 95°C for initial denaturation, followed by 30 cycles of 30 s at 95°C for denaturation, 45 s at 55°C for annealing, and 2 min at 72°C for extension, and a final 5 min cycle at 72°C for the final extension. The putative function of the disrupted gene was determined by BlastP and conserved domain searches with the translated amino acid sequence.

### Assessing polar effects of Tn*5* mutagenesis

The expression of genes flanking the mini-Tn*5* insertion site in RS47-2 was assessed using two-step reverse transcription PCR (RT-PCR). Total RNA was extracted using a RNeasy^®^ PowerMicrobiome^™^ kit (Qiagen) as per manufacturer’s instructions, including the addition of 100 μL 25:24:1 (vol/vol/vol) phenolchloroform-isoamyl alcohol to the PowerBead tube prior to the addition of the sample. Total RNA was then used as a template for cDNA synthesis using the QuantiTect^®^ Reverse Transcription (Qiagen) kit. RT-PCR reactions were carried out in a total volume of 25 μL using the Qiagen Taq core kit according to manufacturer’s instructions, with 20 ng total DNA template and 15 pmol of each primer pair. The following primer pairs were used for each flanking gene assessed respectively: GENE-B-F (5′-GCCATCAGAGCAATTCGTGC-3′) and GENE-B-R (5′-ACCTGCCATCACGGCAAATA-3′); GENE-D-F (5′-TCGGGACTACTTGGGCCATA-3′) and GENE-D-R (5′-ACGCGACATTAGCCCAGAAA-3′). Standard DNA extractions were used as positive controls, and reactions with no template DNA or no reverse transcriptase were utilized as controls for gDNA contamination.

### Analysis of LPS structure

Overnight cultures were pelleted by centrifugation (2 min, 13000 ×*g*) and washed once in PBS. The washed cell pellet was then resuspended in 500 μL of PBS, before addition of 250 μL LPS buffer 1 (0.1875 M Tris pH 6.8, 6 % SDS, 30 % glycerol). The solution was then boiled for 5 min, before being cooled to room temperature. Once cooled, 10 μL of the boiled solution was mixed with 35 μL of LPS buffer 2 (0.0625 M Tris pH 6.8, 0.1 % SDS, 10 % glycerol, 0.1 % bromophenol blue), and 12.5 μL of proteinase K (20 mg mL^−1^, Fisher). The resulting mixture was incubated at 55°C for 14 h. The prepared LPS lysate was then run on a 10 % Tris-glycine gel (Biorad) before incubation in fixing solution 1 (40 % ethanol, 5 % acetic acid) overnight at room temperature on an orbital shaker at 60–70 rpm. After incubation, fixing solution 1 was replaced with fixing solution 2 (0.7 % periodic acid, 40 % ethanol, 5 % acetic acid) and the gel was incubated at room temperature for 5 min on an orbital shaker at 60–70 rpm. The gel was initially rinsed with deionized water, then washed in 1000 mL of deionized water for 15 min on an orbital shaker at 60–70 rpm. This wash step was repeated a further 2 times, before the gel was silver stained as described by Tsai and Frasch (1982).

### Hydrophobicity assays

Overnight cultures were pelleted by centrifugation and the supernatant discarded. Cell pellets were then washed with 5 mL of phosphate urea magnesium (PUM) buffer (pH 7.1, K_2_HPO_4_.3H_2_O; 22.2 g L^−1^, KH_2_PO_4_; 7.26 g L^−1^, CH_4_N_2_O; 1.8 g L^−1^, MgSO_4_.7H_2_O; 0.2 g L^−1^) before pelleting by centrifugation. This wash step was repeated three times before the remaining cell suspension was adjusted to an OD _400 nm_ of 1.0 ± 0.1. The washed bacterial suspension was then separated into 2 mL aliquots and added to sterile glass test tubes (15 mm diameter) with 400 μL of hexadecane (ACROS Organics^™^). Test tubes were vortexed for 30 s before being incubated at 37°C for 30 min. Following incubation, OD _400 nm_ readings were taken from the lower aqueous phase, and cell surface hydrophobicity was calculated using the following equation (Rosenberg et al., 1983; Wilson-Nieuwenhuis et al., 2017; Slate, 2019):


$$\mathrm{Percentage Adhesion}\;\left(\%\right)=\left(\frac{1-A}{A_{O}}\;\right)\times 100,$$

where, A_O_ is the OD_400 nm_ before the addition of hexadecane and A is the OD_400 nm_ from the lower aqueous phase, following the addition of the hexadecane. The hydrophobicity of different isolates can be scored as follows: < 20 %—hydrophilic, 20–50 %—moderately hydrophobic, > 50 %—strongly hydrophobic.

### Evaluation of cell autoaggregation

Percentage autoaggregation assays were performed as described previously by Rahman et al. (2008) and Wang et al. (2012b), with minor alterations (Rahman et al., 2008; Wang et al., 2012b). Overnight cultures were washed twice in PBS and normalized to OD_600 nm_ 1.0 ± 0.1 in a total volume of 5 mL. The top 0.5 mL of culture was removed and OD_600 nm_ was measured before the culture was incubated statically at room temperature for 2 h. Following incubation, OD_600 nm_ of the top 0.5 mL of culture was measured for a final time and the percentage change in OD_600 nm_ was measured to calculate percentage autoaggregation.

### Complement-mediated bactericidal activity assay

Normal human serum (NHS) was prepared as described by Laabei et al. (2018). Bacterial cultures were washed in PBS and diluted in Gelatin Veronal Buffer (GVB^++^; NaCl; 4.2 g L^−1^, Na-barbital; 0.185 g L^−1^, Barbituric acid; 0.286 g L^−1^, Gelatin; 1 g L^−1^, MgCl_2_; 1 mM, CaCl_2_; 0.15 mM) to a final OD_600 nm_ of 0.1. The normalised bacterial cells were then mixed with 15 %, 30 %, or 50 % NHS and incubated at 37°C for 1 h. Cells mixed with GVB^++^ were used as input controls, and 50 % heat inactivated serum (incubated at 56°C for 30 min) was used as a control. To calculate bacterial survival, aliquots of bacteria were removed at time 0 and following 1 h incubation, serially diluted in PBS, and plated on to MacConkey agar without salt for enumeration of single colonies.

### Complementation of RS47-2 with a functional *smvR* and confirmation of *smvA* repression

A pGEM-T construct containing a functional copy of *smvR* (pGEM-T*smvR*), previously described by Pelling et al., (2019a), and empty pGEM-T vector (pGEM-T*empty*), were introduced to RS47-2 by electroporation (0.1 cm gap cuvettes, 1.25 V, 25 μF, 200 Ω; Pelling et al., 2019a). Transformants were selected for on NSLB agar supplemented with 100 μg mL^−1^ ampicillin and 50 μg mL^−1^ kanamycin. Repression of *smvA* in RS47-2 derivatives harboring pGEM-T*smvR* was confirmed using two-step RT-qPCR. Total RNA was extracted using the RNeasy PowerMicrobiome kit (Qiagen) with minor modifications as detailed in Pelling et al. (2019a). Total RNA was used as the template (1 μg per reaction) for cDNA generation using the Protoscript First Strand cDNA Synthesis Kit according to manufacturer’s instructions. qPCR was carried out using a StepOnePlus^™^ Real-Time PCR System (Applied Biosystems), in total reaction volumes of 20 μL consisting of: Sybr Green qPCR mastermix (10 μL), cDNA template (5 μL), nuclease free water and 10 pmol of the forward primer SMVA-F (5′-TCGCCACCCTTATTGCCATT-3′) and the reverse primer SMVA-R (5′-CGGCGACTAACTGTAAGCGT-3′). Duplicate technical replicates were performed for each of three biological replicates and negative controls included reactions with no template cDNA as well as extracted RNA not subject to reverse transcription to confirm successful gDNA removal. A calibration curve of DNA standards was generated using a pGEM-T plasmid containing a fragment of *smvA* to permit quantification of *smvA* expression.

### Motility assays

Overnight cultures were normalized to OD_600 nm_ 1.0 (± 0.1) in LB broth. To assess swimming motility, 2 μL aliquots of normalized overnight culture were stabbed into the center of a 25 mL 0.3 % LB agar plate and incubated at 37°C for 12 h. To assess swarming motility, aliquots of 10 μL were stabbed into the center of a 25 mL 1.5 % LB agar plate and incubated at 37°C for 18 h. Following incubation, swim and swarm diameters were measured in triplicate using digital calipers (accurate to 0.01 mm, Fisher).

### *In vitro* bladder models

Protocols from Nzakizwanayo et al. (2019) were adapted to provide an *in vitro* model of the catheterized urinary tract. Artificial urine (AU) was prepared as two separate solutions, a 5 × stock solution (11.5 g L^−1^ anhydrous sodium sulfate, 3.25 g L^−1^ magnesium chloride hexahydrate, 23 g L^−1^ sodium chloride, 3.25 g L^−1^ tri-sodium citrate, 0.1 g L^−1^ sodium oxalate, 14 g L^−1^ potassium di-hydrogen orthophosphate, 8 g L^−1^ potassium chloride, 5 g L^−1^ ammonium chloride, 25 g L^−1^ gelatine, 5 g L^−1^ tryptone soy broth; this solution component was autoclaved) and a separate calcium/urea solution (filter sterilized; 0.44 μM filter; 156.25 g L^−1^ urea, 3.0625 g L^−1^ calcium chloride). The 5 X stock solution was pH adjusted to 5.75 using a pH meter (HI 2210; Hanna Instruments). A 1 L volume of the 5 × stock solution was combined with 400 mL of the urea/calcium solution and topped up to 5 L with sterile deionized water, resulting in a final pH of 6.10.

Double-walled glass vessels (*in vitro* bladder models) were maintained at 37°C throughout the experiment using a circulating water bath. Silicone catheters (BARDIA^®^ AQUAFIL^®^ All Silicone Foley Catheter; Bard) were inserted into the inner chamber of the bladder model, and the balloons were inflated using the 10 mL syringe of sterile water provided. A sterile drainage bag (BARDIA^®^ Drainable Bed Bag with 180° lever tap; Bard) was attached to the catheter to create a sterile, closed drainage system. Bladder models were inoculated with 10 mL of OD_600 nm_ 1.0 (± 0.1) normalized culture resuspended in 10 mL of AU (*ca.* 10^8^ CFU mL^−1^) which were allowed to establish for 1 h before the activation of AU flow. For the duration of the experiment, AU was supplied to the model at a constant flow rate of 3 rpm. Exact flow rates for each model were calculated by measuring the amount of residual AU accumulated in the drainage bag in 4 h. Bladder models were sampled from the central chamber for CFU mL^−1^ calculations and pH analysis at time of inoculation (T_0_) and time of blockage (T_f_; *n* = 5).

### Statistical analysis

Statistical analysis was completed in Graphpad Prism version 9.3.1, and t-tests were performed on parametric data to identify statistical significance unless stated otherwise.

## Results

### Selection and initial characterization of mutants with reduced CHD susceptibility

A total of 1,152 RS47 mini-Tn*5* mutants were screened for reductions in CHD susceptibility. Initial first pass high-throughput screens identified 531 mutants that failed to grow at 0.5 × RS47 CHD MIC in agar but showed normal growth on media with no CHD. Subsequent characterization of these mutants, using both agar and broth microdilution MIC analysis, identified a single phenotypically stable mutant designated RS47-2. This mutant exhibited an ≥ 8-fold reduction in CHD MIC in liquid media compared to the RS47 parental isolate (64 μg mL^−1^, compared to ≥ 512 μg mL^−1^). Whole genome sequencing of RS47-2 confirmed a single mini-Tn*5* insertion within an open reading frame (ORF) predicted to encode a putative lipopolysaccharide hepatosyltransferase I protein. The corresponding intact ORF in the RS47 WT genome exhibited greatest similarity to the *waaC* gene from *P. mirabilis* HI4320 (PMI_RS15695; 98 % coverage, 97 % identity, *e* = 0.0). PCR amplification of the *waaC* gene region in the RS47 parental strain and RS47-2 mutant confirmed mini-Tn*5* insertion in this gene in RS47-2. Expression of the ORFs flanking *waaC* was also examined using RT-PCR which ruled out polar effects from the mini-Tn*5* insert in RS47-2.

### Phenotypic characterization of RS47-2

**Figure 1:**
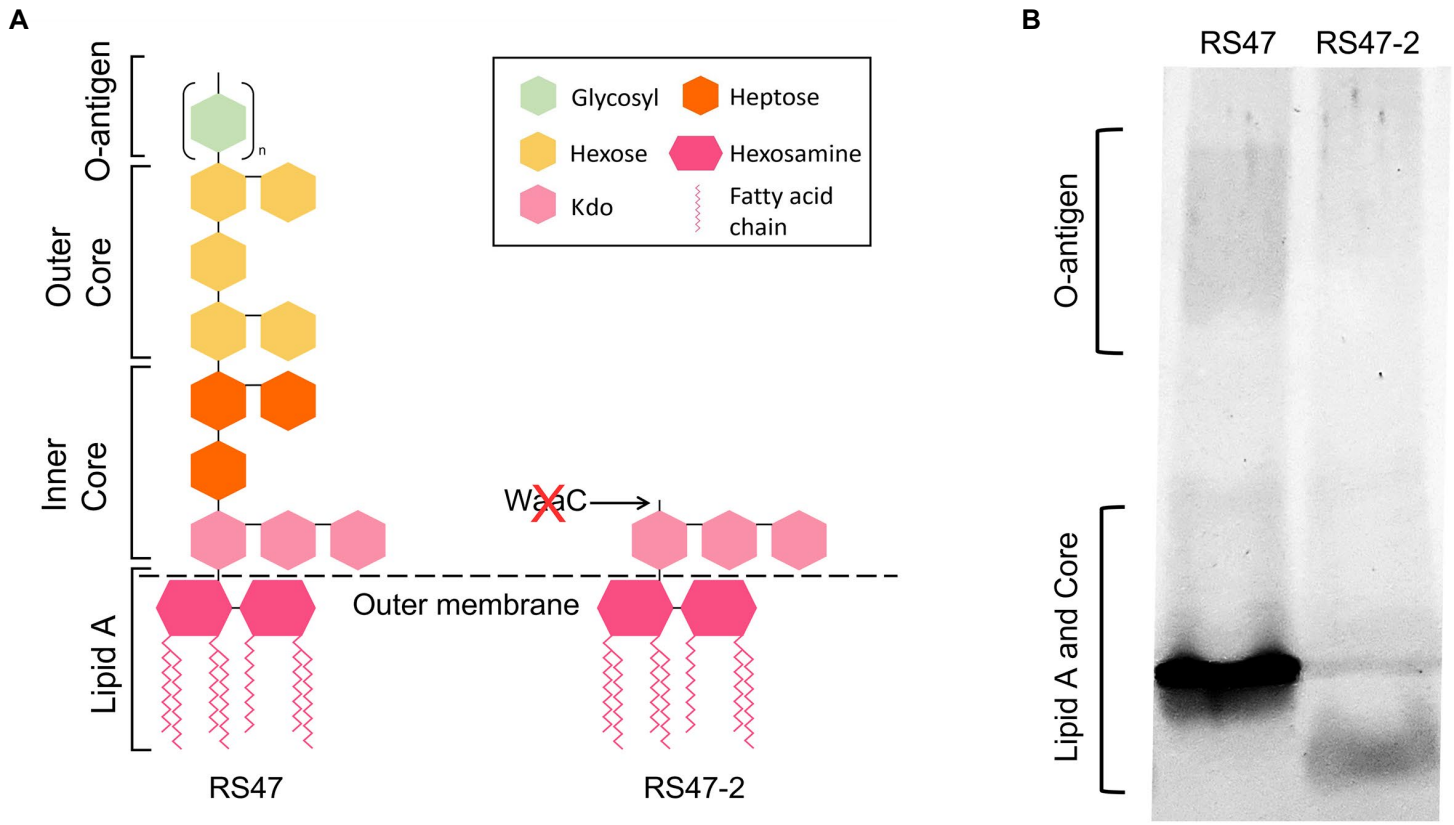
Impact of RS47-2 *waaC* disruption on lipopolysaccharide (LPS) structure. The effects of *waaC* disruption on LPS structure were assessed by comparison of RS47 WT and RS47-2 LPS profiles. **(A)** Illustration showing predicted impact of *waaC* inactivation on LPS structure in RS47-2. **(B)** Separation of LPS lysates on tris-glycine gels comparing core and O-antigen regions between RS47 WT and RS47-2.

WaaC has been described as essential for synthesis of the LPS inner core in other species, and its inactivation associated with a truncated LPS lacking O-antigen (Pearson et al., 2008; Wang et al., 2014). Loss of O-antigen has also been associated with changes to a range of cell surface associated characteristics relevant to antimicrobial susceptibility and virulence, such as hydrophobicity, autoaggregation, human serum susceptibility, and motility (Rowley, 1968; Gygi et al., 1995; McCoy et al., 2001; Morgenstein et al., 2010; Luke et al., 2010a; Nakao et al., 2012; Hu et al., 2021; Wang et al., 2021; Liu et al., 2022). To confirm the predicted impact of *waaC* disruption in RS47-2, we compared the LPS structure and associated characteristics of this mutant to the RS47 parental WT. Loss of O-antigen and clear differences in structure of the inner core region were evident in RS47-2 compared with the WT, congruent with the predicted effects of *waaC* inactivation (Figure 1).

**Figure 2:**
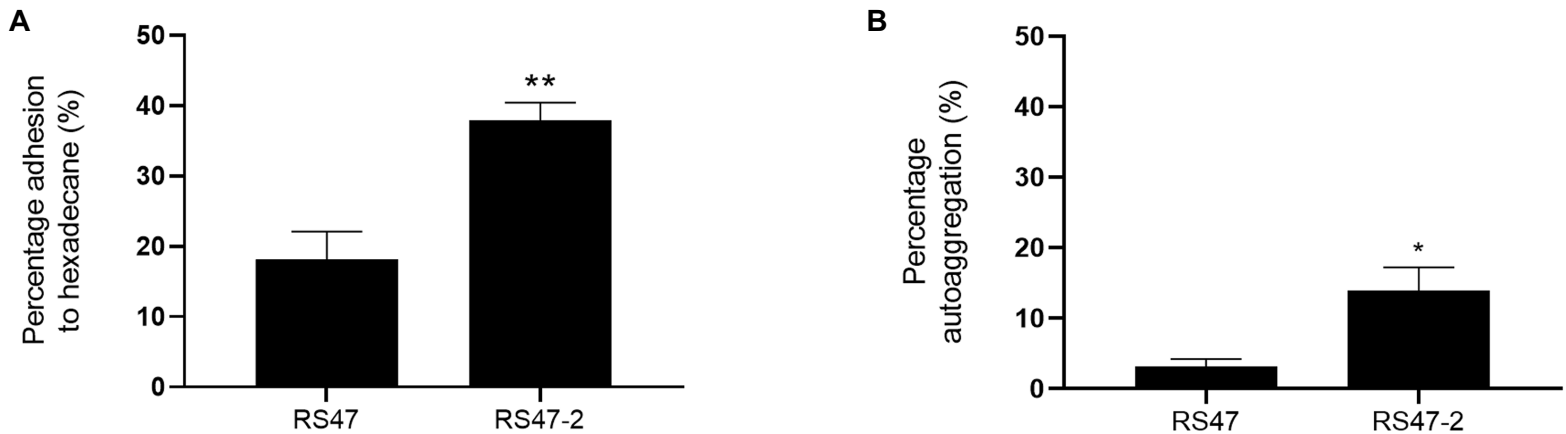
Impact of LPS alterations on hydrophobicity and autoaggregation in RS47-2. **(A)** The hydrophobicity of *Proteus mirabilis* clinical isolate RS47 WT and RS47-2 was evaluated by measuring percentage (%) adhesion to hexadecane. **(B)** Autoaggregation of RS47 WT and RS47-2 cells after 2 h incubation at room temperature. All data represent the mean of 3 biological replicates. Error bars show standard error of the mean (SEM). ^*^p ≤ 0.05. ^**^p ≤ 0.01.

As the O-antigen of the LPS is known to contain negatively charged regions, LPS truncation can also result in increased cell surface hydrophobicity and autoaggregation of cells (Hu et al., 2021; Wang et al., 2021). Hexadecane adhesion assays showed the hydrophobicity of RS47-2 was significantly altered compared to RS47 (Figure 2A). The proportion of hexadecane adhesion indicated RS47-2 to be moderately hydrophobic (37.91 % adhesion), whereas the RS47 parental strain was categorized as hydrophilic (18.17 % adhesion). A moderate but significant increase in autoaggregation was also observed in RS47-2 compared with the wild-type RS47 (Figure 2B).

**Figure 3:**
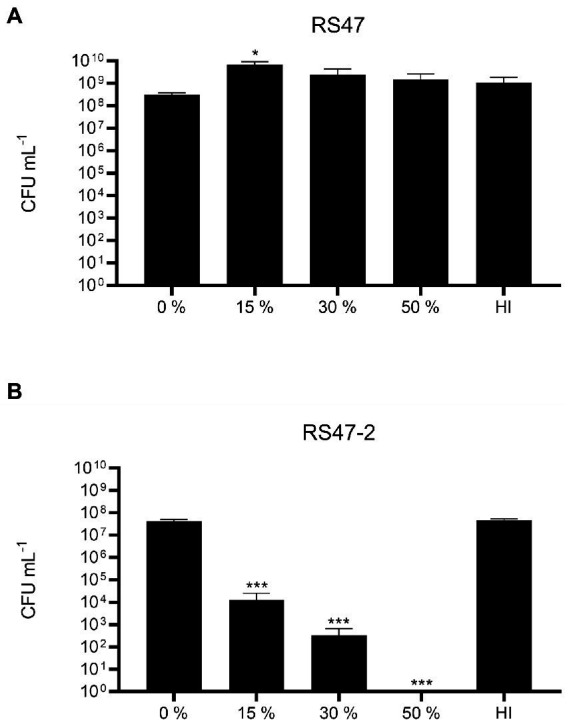
Impact of LPS alterations on survival in NHS. **(A)** Survival of WT RS47 in normal human serum (NHS). **(B)** Survival of RS47-2 in NHS. Heat inactivated serum (HI) was included as a control. Figures show the mean of three biological replicates. Error bars show standard error of the mean (SEM). Ordinary one-way ANOVA with Dunnett’s *post hoc* test were performed, comparing bacterial survival in NHS to the 0 % serum input control. ^*^p ≤ 0.05. ^***^p ≤ 0.001.

**Figure 4:**
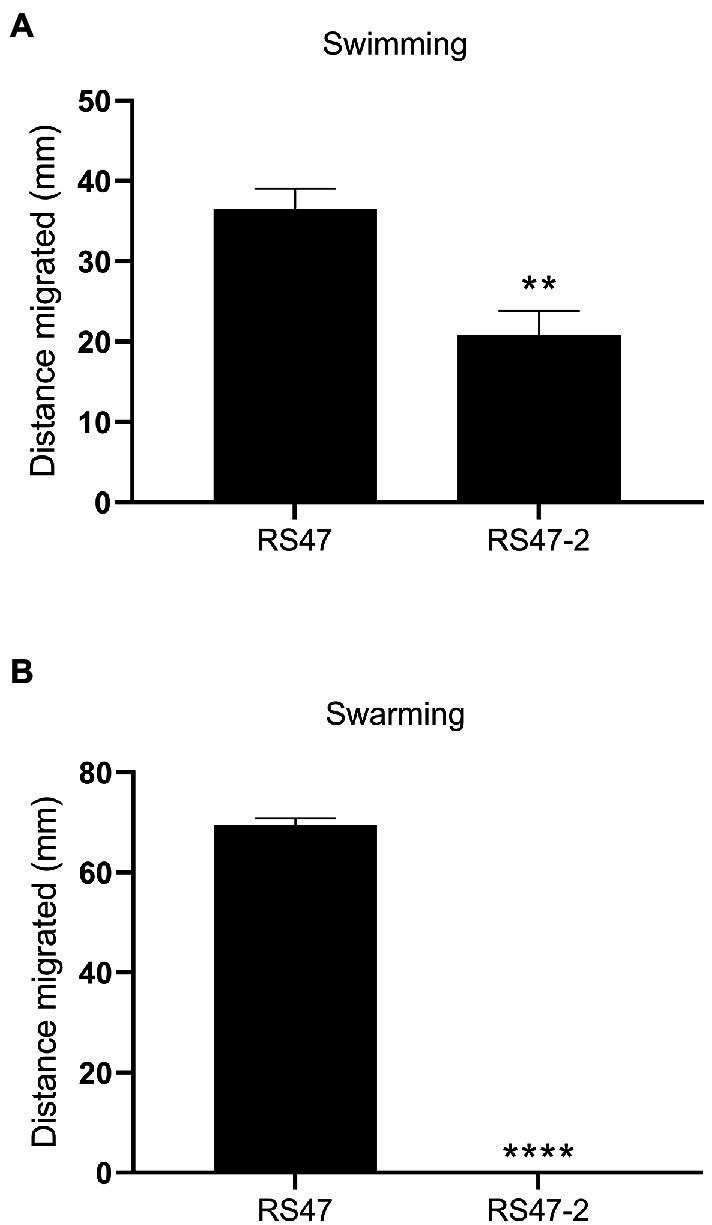
Impact of LPS alterations on motility. **(A)** Swimming ability of RS47 WT and RS47-2 in 0.3  % LB agar. Data shows distance migrated after 12 h incubation. **(B)** Swarming ability of RS47 WT and RS47-2. Data presented shows the diameter of swarm migration over 1.5  % LB agar from the central inoculum after 18 h incubation. A distance of zero mm indicates no expansion of the colony beyond the initial inoculum and no detectable swarming motility. All data represent the mean of five biological replicates and error bars show standard error of the mean (SEM). ^**^p ≤ 0.01; ^****^p ≤ 0.0001.

Also in keeping with defects in LPS production and loss of O-antigen in RS47-2 was the significant increase in serum susceptibility exhibited by this mutant compared to the RS47 WT (Figure 3), as previous studies have shown that LPS truncation results in reduced survival in NHS (Rowley, 1968; Luke et al., 2010b). Swimming motility was significantly reduced in RS47-2 compared with the wild type (Figure 4A) while no swarming motility was evident in RS47-2 following 18-h incubation at 37°C (Figure 4B). Collectively these data supported the predicted disruption of LPS biosynthesis and loss of O-antigen in RS47-2 through inactivation of *waaC*.

### Relative contribution of *smvA* efflux and LPS modifications to CHD susceptibility

We have previously shown that de-repression of the *smvA* efflux system, through inactivation of the *smvR* repressor, is an important factor in the reduced CHD susceptibility of RS47 (Pelling et al., 2019a). To confirm the contribution of changes in LPS structure to increased CHD susceptibility in RS47-2, we restored SmvR activity in both the RS47-2 mutant and RS47 WT (by complementation with a functional copy of the *smvR* gene), and measured impact on CHD susceptibility (Table 2). qPCR assays confirmed that *smvA* expression was significantly reduced in complemented strains compared to those harboring empty vector (≥ 10-fold reduction, *p* ≤ 0.005). In the RS47 WT background where LPS structure was unaltered, repression of *smvA* reduced CHD MIC by at least 2-fold (Table 2). Conversely, LPS changes observed in RS47-2 reduced CHD MIC by at least 8-fold (Table 2). Repression of *smvA* activity in the RS47-2 background, combining loss of SmvA efflux and changes to LPS structure, further reduced CHD MICs by at least 32-fold (Table 2). Collectively these data show that both SmvA mediated efflux and aspects of LPS structure contribute to the CHD tolerant phenotype of RS47.

**Table 2:** Relative contribution of *smvA* efflux and LPS alterations to CHD susceptibility in RS47-2.

Isolate	Characteristics^a^	CHD MIC (μg mL ^−1^)
RS47	smvA ON; waaC ON	> 512
RS47::pGEM-Tempty		> 512
RS47::pGEM-Ts*mvR*	smvA OFF; waaC ON	256
RS47-2	smvA ON; waaC OFF	64
RS47-2::pGEM-Tempty		64
RS47-2::pGEM-Ts*mvR*	smvA OFF; waaC OFF	16–32

a The relevant characteristics of isolates and derivatives are summarized to facilitate interpretation of data: *smvA ON*—*smvA* efflux system is overexpressed; *smvA OFF*—*smvA* efflux system is repressed through complementation with functional s*mvR*; *waaC ON*—no known disruption to gene activity; *waaC OFF*—gene has been inactivated by mini-Tn*5* insertion.

### Impact of *waaC* disruption on susceptibility to biocides and antibiotics

Due to the role of LPS and efflux in limiting the entry of numerous antimicrobial agents into Gram negative cells, the impact of *waaC* inactivation and the contribution of *smvA* efflux activity to the wider antimicrobial susceptibility profile of RS47 was assessed (Table 3). Derivatives of RS47 and RS47-2 carrying empty pGEM-T vector showed no differences in susceptibility to antimicrobials tested compared to parental strains (Table 3). No notable differences in antibiotic susceptibility were observed between RS47 and RS47-2, or in complemented derivatives where *smvA* activity was repressed (Table 3). However, compared to the RS47 WT a significant reduction in RS47-2 MIC was observed for all biocides assayed, with MICs ranging from 4-fold to 64-fold lower than RS47. Furthermore, repression of the *smvA* efflux system in RS47-2 in conjunction with inactivation of *waaC* (RS47-2::pGEM-T*smvR*), resulted in further increases in biocide susceptibility, with biocide MICs between 16- to 256-fold lower than for the RS47 wild-type (Table 3).

**Table 3:** Minimum inhibitory concentrations (MICs) values of various biocides and antibiotics for wild-type, mini-tn5 mutants and complemented isolates (*n* = 3).

	MIC (μg mL^−1^)^a^					
Isolate^b^	RS47	RS47::*smvR*	RS47-2	RS47-2::*smvR*	RS47::empty	RS47-2::empty
	*smvA ON*	*smvA OFF*	*smvA ON*	*smvA OFF*	*smvA ON*	*smvA ON*
	*waaC ON*	*waaC ON*	*waaC OFF*	*waaC OFF*	*waaC ON*	*waaC OFF*
Biocides^c^						
CHD	> 512	**256**	**64**	**16**–**32**	> 512	64
OCT	16	**2**–**8**	**2**	**0.5**–**1**	8–16	2
BZK	64	**16**	**16**	**4**	64	16
CPC	256	**16**	**4**	**2**	256	2
HDPCM	512	**16**	**4**	**2**	256–512	4
Antibiotics^d^						
PMB	> 2048	> 2048	> 2048	> 2048	> 2048	> 2048
NAL	4–8	4	2–4	2	4	2
GEN	8	4	4	4	4–8	4
CHL	8–16	8	8	8	8	8
TMP	8	2–8	4–8	4–8	2–4	4–8
AMX	4	^*^	2	*	*	*

a A > 2-fold difference in MIC compared with the RS47 WT is considered significant and relevant values are highlighted in bold.

b The relevant characteristics of isolates and derivatives are summarized to facilitate interpretation of data: *smvA ON*—*smvA* efflux system is overexpressed; *smvA OFF*—*smvA* efflux system is repressed through complementation with functional s*mvR*; *waaC ON*—no known disruption to gene activity; *waaC OFF*—gene has been inactivated by mini-Tn*5* insertion.

c Biocides tested: CHD, chlorhexidine digluconate; OCT, octenidine dihydrochloride; BZK, benzalkonium chloride, CPC, cetylpyridinium chloride; HDPCM hexadecylpyridinium chloride monohydrate.

d Antibiotics tested: PMB, polymyxin B sulfate; NAL, nalidixic acid; GEN, gentamicin; CHL, chloramphenicol; TMP, trimethoprim; AMX, amoxicillin.

* The MIC for amoxicillin was not calculated for isolates harbouring smvR constructs that encode ampicillin resistance.

### Impact of *waaC* disruption on crystalline biofilm formation

**Figure 5:**
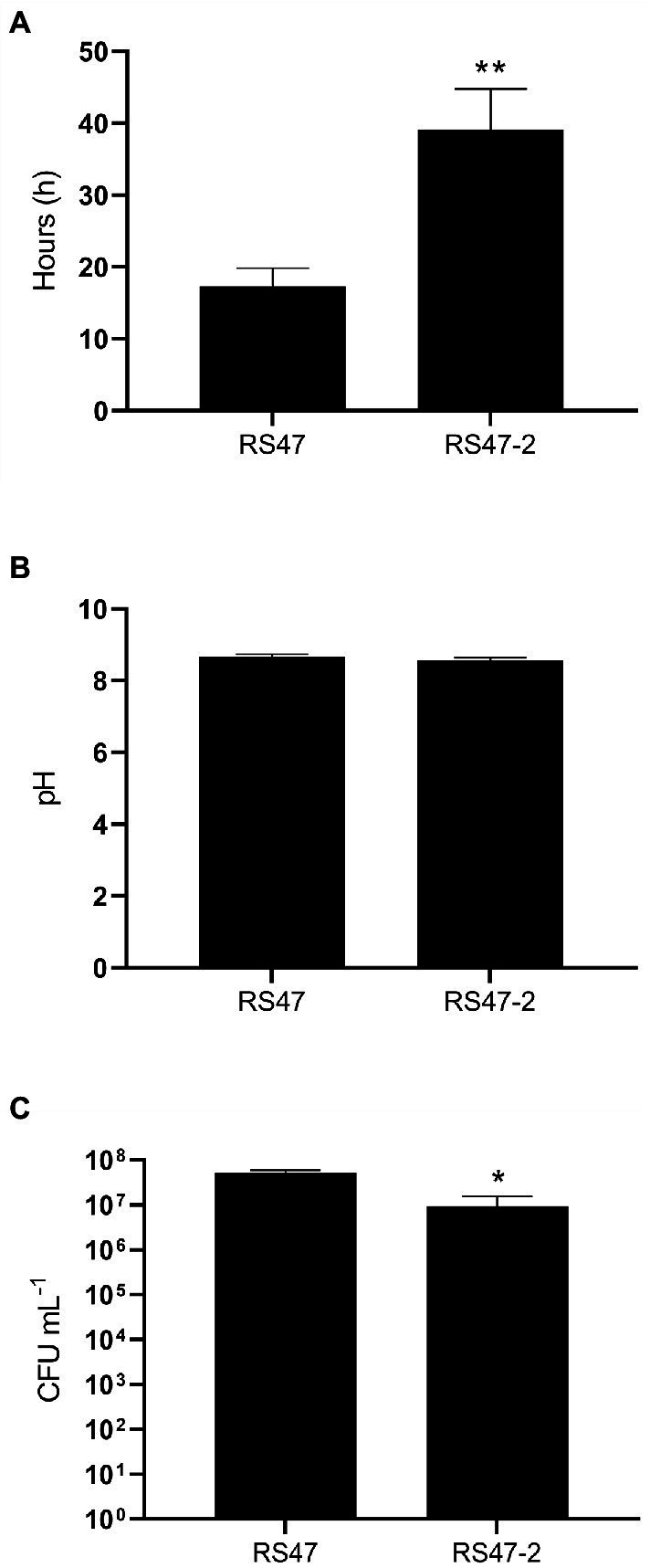
Impact of *waaC* disruption on crystalline biofilm formation. *In vitro* bladder models were used to simulate infection of the catheterized urinary tract. **(A)** Time taken for catheters to block in models inoculated with the RS47 WT or RS47-2 mutant. **(B)** pH of residual bladder urine at time of catheter blockage**. (C)** Number of viable cells in residual bladder urine at time of catheter blockage. Data represents the mean of a minimum of five biological replicates and error bars represent SEM. ^*^p ≤ 0.05; ^**^p ≤ 0.01.

The major clinical complications of *P. mirabilis* CAUTI stem from the blockage of urethral catheters by crystalline biofilm formation. To understand if this aspect of *P. mirabilis* virulence was modulated in RS47-2, *in vitro* bladder models were used to compare crystalline biofilm formation in the RS47 WT and RS47-2 mutant (Figure 5). In these models, RS47-2 took significantly longer to block catheters, indicating that crystalline biofilm formation is attenuated in this mutant (Figure 5A). However, although pH of urine at time of blockage was comparable between RS47 and RS47-2, a small but significant reduction in viable cell numbers was observed for RS47-2 at the time of catheter blockage (Figures 5B,C).

## Discussion

Although our previous work highlighted the key role of *smvA* mediated efflux in reducing *P. mirabilis* susceptibility to CHD (Pelling et al., 2019a), here we show that LPS structure also significantly contributes to the highly CHD tolerant phenotype observed in some *P. mirabilis* clinical isolates. Disruption of *waaC* gene in the CHD tolerant isolate RS47 resulted in a notable increase in CHD susceptibility, regardless of the elevated SmvA efflux activity in this isolate. During LPS biosynthesis, WaaC catalyses the addition of an initial L-glycero-D-manno-heptose molecule to the basal 3-deoxy-d-manno-octulosonic acid-lipid A structure (kdo2-lipid A), to form the LPS inner core (Sirisena et al., 1992; Stojiljkovic et al., 1997; Heinrichs et al., 1998; Grizot et al., 2006; Kanipes et al., 2006; Aquilini et al., 2010; Wang et al., 2016). As such, the inactivation of this gene has been shown to result in the production of a severely truncated LPS lacking inner core heptose residues and O-antigen, and is associated with the “deep rough” phenotype in *E. coli* and other species (Coleman and Deshpande, 1985; Gronow et al., 2000; Pearson et al., 2008; Nakao et al., 2012; Moon et al., 2013; Wang et al., 2014; Bertani and Ruiz, 2018; Lee et al., 2019; Hu et al., 2021).

Phenotypic characterization of the RS47-2 *waaC* mutant also confirmed production of a truncated LPS lacking O-antigen, and with changes to the inner core. In addition, alterations to surface hydrophobicity, autoaggregation, and human serum susceptibility in RS47-2 are also congruent with defects in LPS production and loss of O-antigen (Rowley, 1968; Luke et al., 2010b; Nakao et al., 2012; Hu et al., 2021; Wang et al., 2021; Liu et al., 2022). Because disruption to LPS production has also been associated with changes in motility in *P. mirabilis* (Gygi et al., 1995; McCoy et al., 2001; Morgenstein et al., 2010), we compared both swimming and swarming motility of RS47-2 to the RS47 WT. The reduced swimming and swarming motility observed in RS47-2 are also in keeping with the changes to LPS structure that arise from *waaC* inactivation, and defects in LPS biosynthesis have already been clearly linked with changes to motility in *P. mirabilis* and other species (Belas et al., 1995; Toguchi et al., 2000; McCoy et al., 2001; Inoue et al., 2007; Lindhout et al., 2009; Wang et al., 2016; Lee et al., 2019; Little et al., 2019).

The impact of *waaC* inactivation also clearly altered the overall biocide susceptibility profile in RS47-2, leading to increased susceptibility to a range of cationic biocides in addition to CHD. Similar findings have also been reported for other bacterial species, where deep rough LPS mutants were found to be more susceptible to a range of hydrophobic antimicrobial compounds (Russell and Furr, 1986a; Gilbert et al., 1990; Pagnout et al., 2019). Such observations have already highlighted the important role of LPS in modulating antibiotic susceptibility in Gram-negative bacteria, where this outer membrane acts as a first line permeability barrier that limits the entry of compounds into the cell, and consequently their interaction with cellular targets (Stickler, 1974; Russell, 1986; Russell and Furr, 1986b; Nikaido, 2001; Krishnamoorthy et al., 2017).

The impact of defects in LPS biosynthesis on CHD susceptibility is also congruent with mechanisms through which CHD and similar biocides are believed to gain entry to Gram negative cells (Stickler et al., 1983; Nikaido and Vaara, 1985; Russell et al., 1987; Tattawasart et al., 2000). The uptake of CHD is thought to be mediated *via* a self-promoted mechanism involving interaction of biocide molecules with elements of the LPS inner core (Denyer and Maillard, 2002; Zorko and Jerala, 2008). Although *waaC* inactivation is also predicted to impact inner core synthesis by preventing addition of heptose units, the RS47-2 mutant would be expected to still generate the basal kdo2-lipid A structure (Gronow and Brade, 2001; Raetz and Whitfield, 2002). However, densely packed hydrophilic O-antigen chains found in intact LPS are thought to impede the interaction of CHD molecules with the LPS inner core (Denyer and Maillard, 2002). The O-antigen in *P. mirabilis* may also impede entry of cationic compounds due to the charge of this structure, which could serve to trap cationic compounds in this outer leaflet *via* electrostatic interaction (Tattawasart et al., 2000; Torzewska et al., 2003). Therefore, the loss of O-antigen in RS47-2 likely provides greater exposure of the LPS inner core, potentially facilitating interaction with positively charged amphiphilic CHD molecules, and increasing the uptake of this biocide into the periplasmic space (Denyer and Maillard, 2002; Zorko and Jerala, 2008).

Conversely, mechanisms that modify LPS charge have also been proposed to afford protection against CHD in *P. mirabilis* and other Gram negative species, and are already known to promote resistance to cationic antimicrobial peptides such as polymyxin B (PMB; Olaitan et al., 2014a, 2014b; Krishnamoorthy et al., 2017; Wand et al., 2017). In *P. mirabilis*, the two-component system RppAB regulates the decoration of Lipid-A with 4-amino-4-deoxy-L-arabiose (L-Ara4N) moieties in response to PMB, which increases the net positive surface charge (Kaca et al., 1990; McCoy et al., 2001;Wang et al., 2008; Jiang et al., 2010). This confers resistance to PMB by electrostatic repulsion, and it has been suggested that this mechanism also affords some protection against other cationic antimicrobials such as CHD (McCoy et al., 2001; Wang et al., 2008; Jiang et al., 2010; Krishnamoorthy et al., 2017). In keeping with this theory, *P. mirabilis* isolates with the greatest susceptibility to CHD (8–16 μg mL^−1^) have been found to have mutations in the *rppA* regulator predicted to inhibit its function and have notably lower PMB MICs (Wang et al., 2008; Jiang et al., 2010). Similar mechanisms leading to L-Ara4N decoration of Lipid-A and colistin resistance have been observed following adaptation of *Klebsiella pneumoniae* to CHD (Wand et al., 2017).

This raises the possibility that *waaC* inactivation and the resulting defects in LPS production may perturb this process in *P. mirabilis*, leading to the increased CHD susceptibility observed in RS47-2. Congruent with this hypothesis are previous observations from other species showing that mutations in *waaC* and genes related to LPS core synthesis were associated with increased PMB susceptibility (Kong et al., 2011; Lee et al., 2019). However, in the case of RS47-2, susceptibility to PMB is unaffected by *waaC* disruption and LPS truncation, and no mutations predicted to cause loss of function are evident in the *rppAB* system in the RS47 parental strain. Collectively, these data demonstrate that the increased susceptibility to CHD in RS47-2 is unlikely to be related to perturbation of L-Ara4N Lipid-A decoration. Nevertheless, this does not exclude the possibility that upregulation of this, and other pathways leading to increased net positive surface charge, also contribute to the CHD susceptibility profile in some strains of *P. mirabilis*, and that inactivation of relevant pathways in the RS47-2 mutant may further reduce CHD MIC (Wang et al., 2008; Jiang et al., 2010).

In this context, it is also notable that inactivation of *waaC* did not reduce the RS47-2 CHD MIC to values comparable to those observed in the most CHD susceptible *P. mirabilis* isolates characterized to date (Stickler, 1974; Pelling et al., 2019a; Leshem et al., 2022). Although the RS47-2 CHD MIC was reduced ~8-fold compared to the RS47 parental WT, this still remained 8-fold higher than the lowest CHD MICs we have observed in our isolates (64 μg mL^−1^ in RS47-2 compared to 8–16 μg mL^−1^ for the most susceptible isolates). In the case of RS47-2 this can be explained by the continued overexpression of the *smvA* efflux system in this mutant. Repression of *smvA* activity, in both the RS47-2 mutant and the parental RS47 WT, confirmed the relative contributions of both *smvA* efflux and LPS mediated exclusion to the overall CHD tolerant phenotype of RS47.

**Figure 6:**
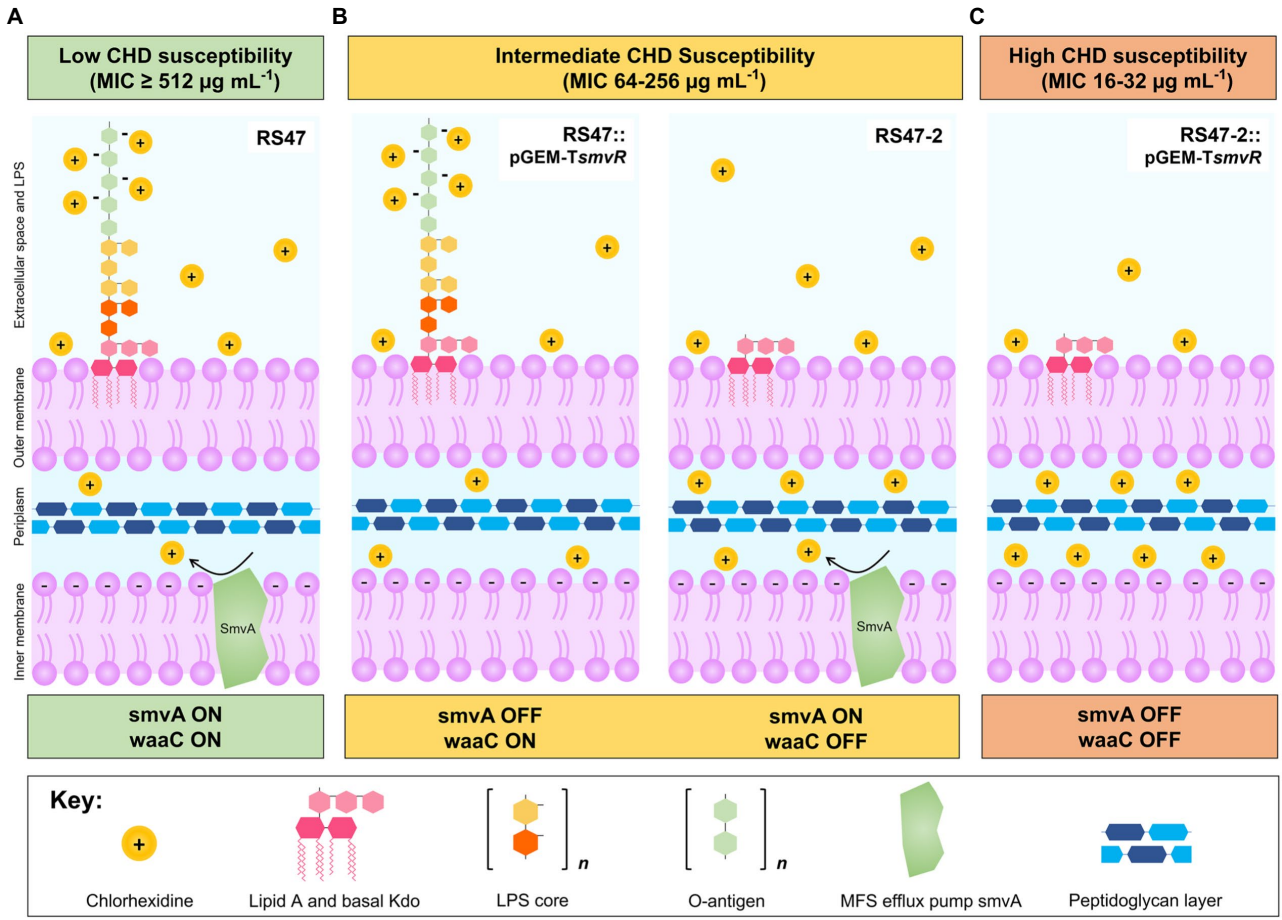
Overview of mechanisms modulating CHD susceptibility in *Proteus mirabilis*. **(A)** *Low susceptibility* to CHD is conferred by LPS structure and *smvA* efflux in WT *P. mirabilis* clinical isolate RS47 (*smvA* on, *waaC* on). Positively charged CHD molecules are occluded by negative charges on the LPS while *smvA* mediated efflux provides further protection from CHD that enters the periplasmic space. **(B)** *Intermediate susceptibility* to CHD is conferred by variation in LPS structure or inactivation of *smvA* efflux activity, observed in RS47::pGEM-T*smvR* (*smvA* off, *waaC* on) and RS47-2 (*smvA* on, *waaC* off) respectively. Intact LPS in RS47::pGEM-T*smvR* occludes entry of CHD into the periplasmic space but subsequent protection from *smvA* efflux is absent. Truncation of LPS in RS47-2 increases penetration of CHD into periplasmic space, but some protection is still provided by *smvA* efflux. **(C)** *High susceptibility* to CHD is observed in RS47-2::pGEM-T*smvR* where protection from LPS and *smvA* efflux are compromised. LPS truncation allows greater CHD penetration into periplasmic space and protection from *smvA* efflux is absent.

These experiments showed that both mechanisms function in synergy to provide the considerable reduction in CHD susceptibility observed in RS47, with LPS structure most likely limiting the initial entry of CHD into the periplasmic space, whilst *smvA* mediated efflux provides further protection against CHD entering this compartment (Wand et al., 2017, 2019; Pelling et al., 2019a). Commensurately, when either mechanism is individually compromised, we observe a partial reduction of CHD susceptibility, and reduction in susceptibility to the lowest observed MICs in *P. mirabilis* isolates only when both mechanisms are disabled (outlined in Figure 6). In addition, this paradigm also appeared to apply to other biocides tested, although with potential variation in the relative importance of *smvA* efflux and *waaC* inactivation for different agents. This also raises the possibility that variation in LPS structure, particularly within the O-antigen region, may in part explain the relatively wide range (8– ≥ 512 μg mL^−1^) of CHD MICs observed among clinical isolates. In particular, this may be an important mechanism in isolates with intermediate level susceptibilities (*ca.* 32–64 μg mL^−1^) that do not exhibit *smvA* upregulation, and is potentially permissive to acquisition of mutations that further reduce biocide susceptibility. In contrast, it was notable that susceptibility to antibiotics tested was unaffected by either changes to *smvA* expression or inactivation of *waaC*, indicating that these mechanisms specifically reduce biocide susceptibility in *P. mirabilis* but do not contribute to general changes in antimicrobial susceptibility.

The adaptation of bacterial pathogens to tolerate increasing concentrations of some biocides also has the potential to modulate other traits relevant to bacterial virulence, and persistence in the hospital environment (Wand et al., 2015; Stein et al., 2019). In the case of *P. mirabilis*, the formation of crystalline biofilms on urinary catheters represents a major clinical complication of infection with this organism, and factors that impact biofilm forming ability could potentially be of much clinical relevance (Mobley and Warren, 1987; Jacobsen et al., 2008; Stickler, 2008, 2014; Holling et al., 2014; Pelling et al., 2019b). In this regard, the ability of RS47-2 to form crystalline biofilms and block urethral catheters is also of considerable interest. In contrast to the enhanced biofilm forming ability documented for LPS inner core mutants in other species, RS47-2 was significantly attenuated in ability to form crystalline biofilms in our *in vitro* infection models (Mireles et al., 2001; Yi et al., 2004; Nakao et al., 2012; Wang et al., 2012a). In the context of CHD susceptibility and strain to strain variation in LPS structure, these observations could suggest the potential for biocide use to select for *P. mirabilis* strains with greater ability to form crystalline biofilms. However, it is worth noting that *in vitro* bladder models provide an indirect measure of crystalline biofilm formation focused on evaluation of catheter blockage, and more direct measurements will be required to understand the specific role of LPS structure in the formation of these biofilms. Further studies are also required to understand if variation in LPS structure between strains has a notable impact on CHD susceptibility, and if this is significantly correlated with ability to block urethral catheters.

The disruption of *waaC* in RS47-2 provides new insights into the mechanisms underlying crystalline biofilm formation in this species and identifies potentially novel targets for the control of this serious clinical complication. In particular, previous studies have suggested that the O-antigen is a major component in the binding of mineral precipitates during *P. mirabilis* crystalline biofilm formation, which is congruent with the increased time to block catheters observed for RS47-2 (Torzewska et al., 2003). As the inner core of LPS is well conserved between Gram negative species, inhibitors of core biosynthesis enzymes such as WaaC could have a broad-spectrum activity, and are already under active investigation in relation to development of new antibiotics and enhancement of existing agents (de Kievit and Lam, 1997; Heinrichs et al., 1998; Cipolla et al., 2011; Nakao et al., 2012; Erwin, 2016; Alexander Mary et al., 2018; Ho et al., 2018; Zhang et al., 2018; Sader et al., 2018a, 2018b; Simpson and Trent, 2019). The impact of *waaC* disruption on RS47-2 crystalline biofilm formation also suggests these compounds could be effective in the control of catheter blockage, and provide a new approach to tackle this important clinical problem. For example, multivalent glycosylated fullerenes have already been shown to inhibit WaaC in *in vitro* enzymatic assays, and structural studies of the WaaC ternary complex are providing insight into potential new inhibitors of the enzyme (Moreau et al., 2008; Durka et al., 2012; Blaukopf et al., 2018).

In conclusion, this study shows that LPS structure is an important factor in both biocide susceptibility and crystalline biofilm formation in *P. mirabilis*. In terms of biocide susceptibility, SmvA-mediated efflux appears to work in synergy with LPS mediated exclusion of biocides, to modulate overall susceptibility in *P. mirabilis.* Defects in LPS structure that lead to increased CHD susceptibility are also associated with attenuated crystalline biofilm formation, and LPS truncation may inhibit the incorporation of crystalline material into the developing biofilm. These findings point to potential targets for controlling catheter blockage and raise important questions about the selective pressure exerted by increasing biocide use on this important pathogen. Further work in these areas will not only facilitate a greater understanding of how bacteria respond and adapt to increasing use of biocides, but may also provide new approaches to control catheter blockage and tackle this important clinical problem.

## Data availability statement

The datasets presented in this study are deposited in the NCBI online repository. The RS47 genome can be found under the BioProject accession number PRJNA554808. Proteus mirabilis smvA and smvR sequences can also be found at GenBank under accession numbers MN265394 and MN265395, respectively. Further inquiries can be directed to the corresponding author/s.

## Author contributions

BJ, HP, and OC conceived and designed the experiments. OC, HP, GG, TM, JN, AS, VB, ML, and AP performed the experiments. BJ, JN, HP, VB, AS, AP, ML, and OC analyzed the data. BJ, LB, MW, KI, SG, and JS supervised the research. OC and BJ wrote the manuscript. All authors contributed to the article and approved the submitted version.

## Funding

This research was primarily supported by funding from the University of Bath Alumni Fund (studentship awarded to OC). JN is supported by funding from the Wellcome Trust (206854/Z/17/Z), AS is supported by funding from the Dunhill Medical Trust (RPGF1906\171), VB is supported by a studentship from the MRC Biomed DTP (MR/N0137941/1), and HP by an iCASE studentship from the MRC (MR/P015956/1). TM also received funding from the JSPS to conduct work on this study.

## Conflict of interest

The authors declare that the research was conducted in the absence of any commercial or financial relationships that could be construed as a potential conflict of interest.

## Publisher’s note

All claims expressed in this article are solely those of the authors and do not necessarily represent those of their affiliated organizations, or those of the publisher, the editors and the reviewers. Any product that may be evaluated in this article, or claim that may be made by its manufacturer, is not guaranteed or endorsed by the publisher.
